# Supplementary material for: The sanitary indoor environment—a potential source for intact human-associated anaerobes
Source: NPJ Biofilms Microbiomes. 2022 Jun 1;8:44. doi: 10.1038/s41522-022-00305-z (PMC9160270; doi:10.1038/s41522-022-00305-z)
Supplement: Supplementary file 3 — Reporting Summary checklist [file 41522_2022_305_MOESM3_ESM.pdf]

nature portfolio

Corresponding author(s): Univ.-Prof. Dr. Christine Moissl-Eichinger

Last updated by author(s): Mar 28, 2022

Reporting Summary

Nature Portfolio wishes to improve the reproducibility of the work that we publish. This form provides structure for consistency and transparency in reporting. For further information on Nature Portfolio policies, see our Editorial Policies and the Editorial Policy Checklist.

Statistics

For all statistical analyses, confirm that the following items are present in the figure legend, table legend, main text, or Methods section.

n/a	Confirmed	
☐	☒	The exact sample size (*n*) for each experimental group/condition, given as a discrete number and unit of measurement
☐	☒	A statement on whether measurements were taken from distinct samples or whether the same sample was measured repeatedly
☐	☒	The statistical test(s) used AND whether they are one- or two-sided<br>*Only common tests should be described solely by name; describe more complex techniques in the Methods section.*
☒	☐	A description of all covariates tested
☐	☒	A description of any assumptions or corrections, such as tests of normality and adjustment for multiple comparisons
☐	☒	A full description of the statistical parameters including central tendency (e.g. means) or other basic estimates (e.g. regression coefficient) AND variation (e.g. standard deviation) or associated estimates of uncertainty (e.g. confidence intervals)
☐	☒	For null hypothesis testing, the test statistic (e.g. *F*, *t*, *r*) with confidence intervals, effect sizes, degrees of freedom and *P* value noted<br>*Give P values as exact values whenever suitable.*
☒	☐	For Bayesian analysis, information on the choice of priors and Markov chain Monte Carlo settings
☒	☐	For hierarchical and complex designs, identification of the appropriate level for tests and full reporting of outcomes
☒	☐	Estimates of effect sizes (e.g. Cohen's *d*, Pearson's *r*), indicating how they were calculated

Our web collection on statistics for biologists contains articles on many of the points above.

Software and code

Policy information about availability of computer code

Data collection

Fastq sequences were processed, using the QIIME2 v2020.2 platform:
1. DADA2 v2020.2.0 algorithm was used to denoise truncated reads and for generation of amplicon sequence variants (ASV).
2. Taxonomic classification was based on the SILVA v132 database.
3. Manual substraction of contaminations (ASVs) and subsequently of control samples was done in Excel v2013.

Measurement of methane concentrations was done with BACVisSingle v6.1 from the BlueSens gas sensor GmbH.

Data analysis

1. Statistical analysis, including differential abundance testing as well as bar charts, box plot and bubble plot generation, was performed in R v4.0.0+ using the phyloseq 1.38.0+ and ggplot2 3.3.0+ packages.
2. Alpha-, beta-diversity and LEfSe analysis were performed using the Calypso online tool, available at http://cgenome.net/calypso/. Accessed in July 2020.
3. Test for differences in the beta-diversity between sample categories was done in QIIME2 v4.0.0+ using PERMANOVA analysis based on Bray-Curtis distances.
4. Microbial phenotype prediction was done with the BugBase online tool, available at https://bugbase.cs.umn.edu/. Accessed in July 2019.
5. Shared ASV detection and VENN diagram construction was done with the BEG online tool available at http://bioinformatics.psb.ugent.be/webtools/Venn/. Accessed in August 2020.
6. Finally, the phylogenetic tree was generated using the following software: the SILVA SINA alignment tool v1.2.11 for multiple sequence alignment (available at https://www.arb-silva.de/aligner/, accessed in November 2020), BioEdit v7.2 to crop sequences, MEGA7 for tree construction, and iTOL v6.5 for postprocessing.

For manuscripts utilizing custom algorithms or software that are central to the research but not yet described in published literature, software must be made available to editors and reviewers. We strongly encourage code deposition in a community repository (e.g. GitHub). See the Nature Portfolio guidelines for submitting code & software for further information.

Data

Policy information about availability of data

All manuscripts must include a data availability statement. This statement should provide the following information, where applicable:

- Accession codes, unique identifiers, or web links for publicly available datasets
- A description of any restrictions on data availability
- For clinical datasets or third party data, please ensure that the statement adheres to our policy

The datasets supporting the conclusions of this article are available in the European Nucleotide Archive (ENA) repository, Primary Accession: PRJEB41618 in https://www.ebi.ac.uk/. Further details can be found in Supplementary Table 5.

Field-specific reporting

Please select the one below that is the best fit for your research. If you are not sure, read the appropriate sections before making your selection.

☐ Life sciences ☐ Behavioural & social sciences ☒ Ecological, evolutionary & environmental sciences

For a reference copy of the document with all sections, see nature.com/documents/nr-reporting-summary-flat.pdf

Ecological, evolutionary & environmental sciences study design

All studies must disclose on these points even when the disclosure is negative.

Study description	A healthy human microbiome relies on the interaction with and exchange of microbes that takes place between the human body and its environment. People in high-income countries spend most of their time indoors and for this reason, the built environment (BE) might represent a potent source of commensal microbes. Anaerobic microbes are of particular interest, as researchers have not yet sufficiently clarified how the human microbiome acquires oxygen-sensitive microbes.<br>We sampled the bathrooms in ten households and used propidium monoazide (PMA) to assess the viability of the collected prokaryotes. We compared the microbiome profiles based on 16S rRNA gene sequencing and confirmed our results by genetic and cultivation-based analyses. Quantitative and qualitative analysis revealed that most of the microbial taxa in the BE samples are human-associated. Less than 25% of the prokaryotic signatures originate from intact cells, indicating that aerobic and stress resistant taxa display an apparent survival advantage. However, we also confirmed the presence of intact, strictly anaerobic taxa on bathroom floors, including methanogenic archaea. As methanogens are regarded as highly sensitive to aerobic conditions, oxygen-tolerance experiments were performed with human-associated isolates to validate their survival. These results show that human-associated methanogens can survive oxic conditions for at least 6 h.<br>This study enabled us to We collected strong evidence that supports the hypothesis that obligate anaerobic taxa can survive in the BE for a limited amount of time. This suggests that the BE serves as a potential source of anaerobic human commensals.
Research sample	Samples were collected from ten different family houses in the vicinity of Graz, Austria (in March 2017). All houses were occupied by at least one adult and five of the houses by families with children.<br><br>For cultivation experiments, three archaeal type strains including Methanosphaera stadtmanae DSM no. 3091, Methanobrevibacter smithii DSM no. 2375, and Methanomassiliicoccus luminyensis DSM no. 25720 as well as a fresh isolate from human feces Methanobrevibacter sp. (unpublished), were used.
Sampling strategy	Per household, two areas of 30 square centimetre in the proximity of the toilet were cleaned with bleach and sterile water and left uncovered and untouched for 7 days. After 7 days, both areas were sampled using a sterile nylon swab that has been dipped in 0.9% saline (NaCl) solution. Each area was sampled with the swab three times, by rotating the swab every time before sampling the area again.
Data collection	Meta data (including sampling time, number and type of residents, etc. - see Supplementary Table 1 for further information) were recorded by the reasearch team at the time of sampling. gDNA concentration was measured immediately after DNA extraction.
Timing and spatial scale	Sampling of all 10 households was performed between the 27th and 30th of March 2017, 7d after the respective bleach treatment.
Data exclusions	No data was excluded from the indoor samples.<br><br>Few replicates of archaeal cultures that became oxic before the oxigen challenge were excluded from further analysis.
Reproducibility	For oxygen tolerance test of methanogenic archaea we used public available strains and aim to publish the new isolate in the near future. All experiments were performed in triplicates, in which all replicates and time points originated from the same culture.
Randomization	The selection of both locations in close vicinity of the toilet as well as the allocation of the samples into PMA- or untreated group were performed randomly.
Blinding	Blinding is not relevant in our study.

Did the study involve field work? ☒ Yes ☐ No

Field work, collection and transport

Field conditions	All samples were taken in Graz, Austria, within 4 days at the end of March.
Location	All sampling locations were within Graz city at N47.06667, E15.45.
Access & import/export	Samples were collected by our research staff. Access to the flats was granted by the residents and no import/export was neccessary.
Disturbance	No disturbance was caused in this study.

Reporting for specific materials, systems and methods

We require information from authors about some types of materials, experimental systems and methods used in many studies. Here, indicate whether each material, system or method listed is relevant to your study. If you are not sure if a list item applies to your research, read the appropriate section before selecting a response.

Materials & experimental systems

n/a	Involved in the study
☒	☐ Antibodies
☒	☐ Eukaryotic cell lines
☒	☐ Palaeontology and archaeology
☒	☐ Animals and other organisms
☐	☒ Human research participants
☒	☐ Clinical data
☒	☐ Dual use research of concern

Methods

n/a	Involved in the study
☒	☐ ChIP-seq
☒	☐ Flow cytometry
☒	☐ MRI-based neuroimaging

Human research participants

Policy information about studies involving human research participants

Population characteristics	Data from the human microbiome was gathered from other, in-house studies as indicated in Supplementary Table 5.<br>All samples originated from healthy, untreated participants with residency in Styria, Austria.
Recruitment	Participants were recruited by healthcare and research staff of the Medical University Graz.
Ethics oversight	All studies were approved by the ethics committee of the Medical University in Graz.

Note that full information on the approval of the study protocol must also be provided in the manuscript.
